# Fractionation of a tumor-initiating UV dose introduces DNA damage-retaining cells in hairless mouse skin and renders subsequent TPA-promoted tumors non-regressing

**DOI:** 10.18632/oncotarget.6932

**Published:** 2016-01-18

**Authors:** Gerline van de Glind, Heggert Rebel, Marika van Kempen, Kees Tensen, Frank de Gruijl

**Affiliations:** ^1^ Department of Dermatology, LUMC, Leiden, 2333RC, The Netherlands

**Keywords:** UV carcinogenesis, dose fractionation, CPD retaining basal cells, hyperkeratotic tumors

## Abstract

Sunburns and especially sub-sunburn chronic UV exposure are associated with increased risk of squamous cell carcinomas (SCCs). Here we focus on a possible difference in tumor initiation from a single severe-sunburn dose (on day 1, 21 hairless mice) and from an equal dose fractionated into very low sub-sunburn doses not causing any (growth-promoting) epidermal hyperplasia (40 days daily exposure, n=20). From day 47 all mice received 12-O-Tetradecanoylphorbol-13-acetate (TPA) applications (2x/wk) for 20 weeks to promote tumor development within the lifetime of the animals. After the sub-sunburn regimen sparse DNA damage-retaining basal cells (quiescent stem cells, QSCs) remained in the non-hyperplastic epidermis. These cells were forced to divide by TPA. After discontinuation of TPA tumors regressed and disappeared in the ‘sunburn group’ but persisted and grew in the ‘sub-sunburn group’ (0.06 vs 2.50 SCCs and precursors ≥4mm/mouse after 280 days, p=0.03). As the tumors carried no mutations in *p53*, *H/K/N-Ras* and *Notch1/2,* these ‘usual suspects' were not involved in the UV-driven tumor initiation. Although we could not selectively eliminate QSCs (unknown phenotype) to establish causality, our data suggest that forcing specifically DNA damage-retaining QSCs to divide – with high mutagenic risk - gives rise to persisting (mainly ‘in situ’) skin carcinomas.

## INTRODUCTION

The incidence of skin carcinomas has strongly increased since the 1970-s by an average of about 4% per year in NW Europe, to a staggering 100 to 200 new cases per 10^5^ persons per year, despite the temperate climate [1–3]. UV radiation plays an important role in the etiology of skin carcinomas, both basal cell carcinomas (BCCs) and squamous cell carcinomas (SCCs) [4, 5]. Kennedy et al. [6] showed that sunburns as well as chronic sun exposure are associated with an increased risk of developing SCCs.

UV radiation preferentially damages sites of neighboring pyrimidine bases in a DNA strand, forming dimers between these bases: either cyclobutane pyrimidine dimers (CPDs) or 6-4 photoproducts (6-4PPs) [7, 8]. These photo lesions cause a distortion of the DNA helix, rendering DNA polymerase unable to read the DNA template, which may result in cytosine (C) → thymidine (T) at sites of adjacent pyrimidines and CC→ TT transitions. These specific transitions are considered UV signature mutations [9, 10]. In SCCs and BCCs the *P53* tumor suppressor genes bear these UV signature mutations [10, 11]. A suitable experimental model for SCCs has been established by chronic (daily) exposure of hairless mice to UV radiation [12]; here the *p53* gene is targeted leading to the UV signature mutations [13], in line with human SCCs. In contrast to two-stage chemical skin carcinogenesis [14], the UV tumors bear no *H-Ras* mutations [15]. Our group found that microscopic foci with overexpression of p53 protein in mutant conformation (detected with antibody PAb240) were already present in UV-exposed mice long before the tumors appeared [16–18]. Such ‘p53 patches’ have also been found in sun-exposed human skin, which after micro-dissection were found to harbor UV-signature mutations in *P53* genes [19, 20].

The essential cancer traits appear to develop in stages in the neoplastic cell [21], which takes time: cancer is therefore predominantly a disease of the elderly. Hence, the targeted cell in carcinogenesis needs to reside in the body for a long time in order to acquire a series of alterations (gene mutations) resulting in malignant growth. Furthermore, the cell probably needs to divide for fixation of mutations (most likely to arise in replication of damaged DNA) and to pass on the mutations to daughter cells. These requirements of long term residence combined with cell division point at stem cells as likely targeted candidates [22, 23].

Mitchell et al. [24] found that CPDs accumulated in some cells located in the basal layer of the epidermis in the course of chronic low level UV radiation and these cells retained CPDs long after discontinuation of UV exposures (> 50 days). Such cells were found to occur in human skin too [25]. If UV exposure caused significant epidermal hyperplasia (e.g. after a sunburn dose), the CPD-retaining epidermal cells were no longer observed [25]. Our group confirmed that at low level chronic UV exposure (1/7 of the threshold dose for a sunburn/day for 40 days) in absence of hyperplasia CPDs accumulated in long-residing non-dividing skin cells, i.e., in some basal cells and in fibroblasts in the upper dermis. And we went on to show that persistent quiescent (BrdU-retaining) epidermal stem cells in the basal layer accumulated and retained UV-induced CPDs while neighboring cells did not (because of cell division and epidermal turnover) [26]. The CPD-retaining basal cells (CRBCs) were found to be repair proficient, since 6-4PPs did not accumulate [26]. CRBCs could be forced to proliferate by application of 12-*O*-Tetradecanoylphorbol-13-acetate (TPA) while the CPD-retaining fibroblasts showed no apparent reaction [25, 27]. With repeated TPA applications the accumulated DNA damage in CRBCs was gradually dispersed over daughter cells, and the CRBCs disappeared.

As mentioned above, both sunburns and chronic (sub-sunburn) UV exposure play a role in SCC development in humans [6] but the life-long accumulated UV dose as from chronic (sub-sunburn) exposure appears to be the major exogenous risk factor [28]. Considering also that a severe sunburn exposure induces hyperplasia and no CRBCs (> 4 days after exposure [25]) whereas the same dose fractionated in daily exposures of 1/7 of the sunburn threshold dose does induce CRBC and no hyperplasia, we set out to investigate the effect of non-hyperplastigenic sub-sunburn dose fractionation on skin carcinogenesis. To this end, one group of hairless mice (sunburn group n=21) received a single severe sunburn dose on day 1 while another group (sub-sunburn group) received the same total dose in 40 daily fractions (each 1/7 of the sunburn threshold dose) to induce CPD-retaining cells. After one week pause both groups were subsequently treated with TPA for 20 weeks to raise skin tumors within the lifetime of the mice (either a single high UV exposure or tumor promotion alone does not give rise to tumors in hairless mice [29]; further information in Materials and Methods); the time line of the experimental protocol for both groups is given in Supplementary Figure S1.

The tumors were scrutinized for (dis-)similarities with tumors induced by two-stage chemocarcinogensis (mostly papillomas with *H-Ras* mutations instead of SCCs) and skin carcinogenesis by chronic UV exposure (i.e. life-long daily sub-sunburn exposure resulting in SCCs and actinic keratosis as benign precursors, both types of lesions with UV-signature mutations in *p53*).

## RESULTS

### CPDs accumulated in isolated epidermal basal cells and in fibroblasts after low dose UV

**Figure 1:**
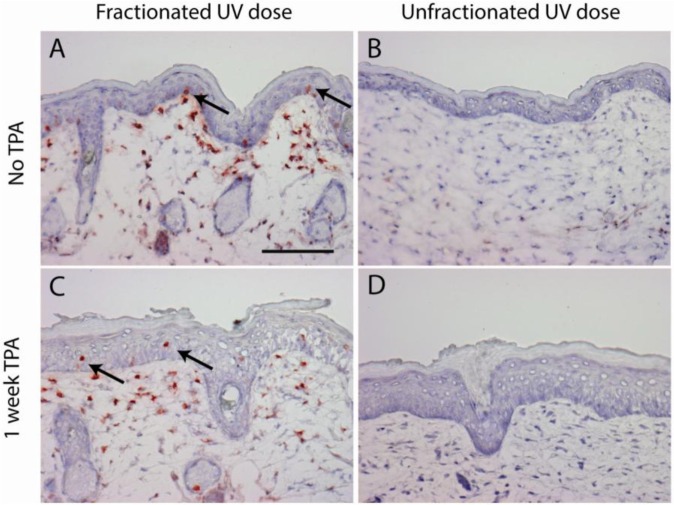
CPD retaining cells are only present after a fractionated sub-sunburn UV dose in epidermis (arrows) and in upper dermis (non-dividing fibroblasts) Mouse skin after fractionated sub-sunburn UV dose before TPA treatment, day 47 **A.** and after TPA treatment, day 54 **C.** and correspondingly after the unfractionated sunburn UV dose **B.** and **D.** Scale bar 100 μm.

As observed in our previous experiment [27], mice in the ‘sub-sunburn group’ that received the low dose fractions of UV spread over 40 days did not show any immediate observable effects, whereas mice in the ‘sunburn group’ that received the unfractionated high dose of UV showed severe sunburn which resolved in 2 weeks. From day 47 onward both groups of mice received dorsal TPA treatment twice a week for 20 weeks. TPA treatment induced epidermal cell proliferation shown by epidermal hyperplasia (Figure 1C+1D) and, as we have shown earlier, an increase in Ki67+ epidermal cells, but not in (CPD-retaining) fibroblasts in the upper dermis [27]. Mice from both groups showed scaling of the skin after TPA treatment. We observed CPD-retaining cells in the sub-sunburn group (Figure 1A). Most of the epidermal CPD-retaining cells were located in the basal layer, i.e., CRBCs, (1.7% of basal cells heavily stained for CPDs). Upon TPA treatment CRBCs were lost and more CPD-containing cells were found suprabasally (Figure 1C, arrows). CPD-retaining cells were also observed in the dermis of these mice (Figure 1A+1C) but these cells showed no apparent reaction to TPA, no cell morphology of proliferation/mitosis or apoptosis (conform our earlier experiment where we stained for Ki67 and activated caspase 3 [27]; these fibroblasts do eventually lose the CPDs apparently by slow repair). We did not observe any CPD-retaining cells in mice that received a single UV overexposure (Figure 1B+1D).

### Fractionated sub-sunburn UV exposure followed by TPA-induced sparse p53 patches

Skin samples were taken after 8 weeks of TPA treatment to check for (mutant) p53-overexpressing foci (‘p53 patches’). Immunohistochemical staining was performed on epidermal sheets using both the CM-5 antibody (detecting wild type and mutant p53) and the PAb240 antibody (detecting only mutant p53). No p53 patches were observed in the sunburn group using either of the antibodies. In the sub-sunburn group we found CM5^+^ patches at a frequency of 3.8 patches/cm^2^ (SEM 1.6), yielding about 1 patch per 5000 CRBCs. Using the PAb240 antibody we found 60% fewer patches expressing mutant-p53 (see Supplementary Figure S2).

### Tumor appearance and pathology

Upon TPA treatment tumors occurred in both groups but after discontinuation of TPA application tumors persisted to grow only in the sub-sunburn group and regressed in the sunburn group.

The histopathological analyses largely confirmed our initial macroscopic observations. The sessile based, endophytically growing tumors, which we provisionally qualified as “non-papilloma” tumors (30% cutaneous horns), were apparently all grades of hyperkeratotic tumors ranging from benign actinic keratosis (AK)-like lesions (18/41 benign) through Bowenoid tumors (carcinomas in situ, 17/41) up to malignant squamous cell carcinomas, SCCs, and 1 basal cell carcinoma, BCC (5/41 malignant); see Table 1 for a comparison with tumor pathology from an older study [30] with chronic, life-long daily UV exposure in which clearly more progression to SCC was observed. In the remainder of this article we will refer to these tumors as AK/SCCs [31]. Only one out of these 41 tumors was not AK/SCC but diagnosed as an inverted papilloma. Exophytically growing tumors (n=7) were confirmed to be papillomas (of which 2 long persisting pedunculated, cauliflower-shaped classical papillomas contained a focus suspect of Bowenoid changes).

**Table 1:** Pathology of tumors (> 3mm) in the present study compared with earlier study with chronic UV exposure (1 MED or 0.03 MED/day), a small percentage of frank papillomas not included; in the present study the mice were either exposed to a single UV exposure of 5.6 MED or a fractionated dose into 0.14 MED/d over a period of 40 days, and after a week subjected to 2 TPA applications per week for a period of 20 weeks

Treatment	SCC (%)	Bowen (%)	AK (%)
1 MED/d chronic (n=68)^1^	73	3	17
0.14 MED/d 40 d + TPA 20 wks (n= 32)	9^2^,^3^	50^4^	34
5.6 MED 1d + TPA 20 wks (n=9)	11^3^	11	78^5^
0.03 MED/d chronic (n=47)^1^	71	9	4

1 De Gruijl et al [30].

2 one BCC excluded.

3 all SCCs from mice sacrificed at the end of the experiment (day 280).

4 2 Bowenoid tumors and 1 AK from mouse sacrificed early (day 224), because of swollen lymph node.

5 5 of 7 AKs from mouse sacrificed early (day 224), because of tumor load and ulceration.

### Tumor-free survival for AK/SCC was significantly shorter after fractionated UV exposure

**Figure 2:**
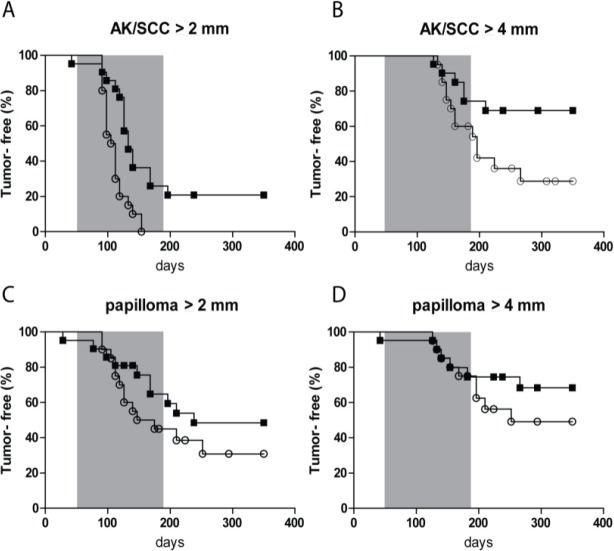
Kaplan-Meier plot of tumor-free fraction of mice for AK/SCC drops significantly faster after fractionated sub-sunburn UV exposure The tumor-free fraction for AK/SCCs ≥2 mm **A.** and ≥4 mm **B.** and papillomas ≥2 mm **C.** and ≥4mm **D.** for mice in the sub-sunburn group (○) or in the sunburn group (■). The grey area in the graphs represent the period in which TPA was applied.

The percentage of tumor-free mice with time is shown in Figure 2 for different threshold sizes of AK/SCCs and for papillomas. For AK/SCC tumors ≥2 mm there was a clear difference between the sunburn and the sub-sunburn group (p=0.0006); all mice in the sub-sunburn group developed a tumor ≥2mm whereas some mice in the sunburn group remained tumor free. Also, for the tumors ≥4mm there was a clear difference between the two groups (p=0.03). More mice in the sub-sunburn group developed AK/SCCs ≥ 4mm. For the papillomas (very small in number) we did not find any significant differences.

Gender differences in UV carcinogenesis have been reported [32], but we found no significant differences in tumor-free survivals between males and females (Supplementary Figure S3).

### Tumor yields showed non-regressing carcinomas only after fractionated UV exposure

**Figure 3:**
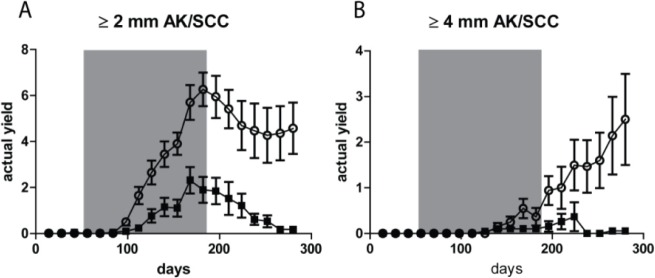
Tumor yields (average number of tumors per mouse) showed non-regressing carcinomas only after fractionated sub-sunburn UV exposure The yield of AK/SCCs ≥2 mm **A.** and ≥4 mm **B.** for mice irradiated with the fractionated sub-sunburn UV dose (○) or with the unfractionated sunburn dose of UV (5.6 MED) (■). The grey area in the graphs represent the period in which TPA was applied. Error bars show SEMs. Virtually all AK/SCCs go into regression in the sunburn group after cessation of TPA applications.

After discontinuation of the TPA treatment AK/SCCs ≥ 2 mm went into regression, as reflected in Figure 3(A) by a drop in tumor yield. Almost all tumors in the sunburn group went into complete regression, while most of the tumors in the sub-sunburn group persisted. For the tumors ≥4 mm we observed an even more distinct difference at day 280. In the sunburn group virtually no AK/SCC grew out to this size, while in the sub-sunburn group these tumors persisted and grew after discontinuation of the TPA regimen (0.06 vs 2.50 AK/SCC ≥4 mm, p=0.03; again, no gender differences, see Supplementary Figure S4).

### No difference in yields of papillomas after different UV treatments

**Figure 4:**
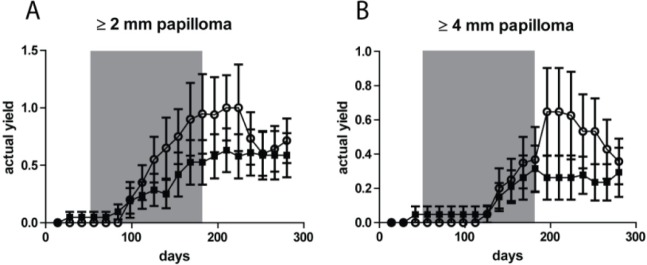
No significant difference in tumor yields (average number of tumors per mouse) for papillomas between the fractionated sub-sunburn and unfractionated sunburn UV dose groups The yield for papillomas ≥2 mm **A.** and ≥4 mm **B.** for mice irradiated with the fractionated sub-sunburn UV dose (○) or with the unfractionated sunburn dose of UV (5.6 MED) (■). The grey area in the graphs represent the period in which TPA was applied. Error bars show SEMs.

Next to AK/SCC tumors we also observed papillomas (growing exophytically) in small numbers. Figure 4 shows the actual yield for papillomas ≥2 mm and ≥ 4mm, respectively. At both thresholds there was no clear difference between the two groups (n.s.). The yield of papillomas was 4-6 fold lower than that of AK/SCCs, with a maximum of 1/mouse for the ≥2 mm tumors and a maximum of 0.6/mouse for the ≥ 4mm tumors, (for AK/SCCs 6 and 2.5/mouse, respectively). In contrast to AK/SCCs papillomas did not appear to regress in the sunburn group after discontinuation of the TPA regimen. We did not find significant differences between male and female mice in papilloma yields (see Supplementary Figure S4).

### Lack of p53, Ras and Notch mutations in AK/SCCs

Samples of tumors (22 AK/SCCs and 3 papillomas), mainly from the sub-sunburn group (17/25), were analyzed for mutations in *p*53 and *Ras* genes (*H-, K-* and *N-Ras*). No mutations were found in *p*53 (exons 4-8). Staining with CM5 showed only sparse cells in the tumor masses overexpressing p53 (except for 1 AK/SCC out of 34 that was abundantly positive but without *p53* mutation). Considering the absence of *p53* mutations this could concern reactive wild-type p53 expression (data not shown). We found two papillomas mutated in *H-Ras* codon 12 (G>A), (1 from each group). Furthermore we found one mutation in *H-Ras* codon 13 (G>T) in an AK/SCC from the sub-sunburn group. No mutations were found in *N-* and *K-Ras.*

In follow-up of this paucity of mutations in *p53* and *RAS*, we decided to investigate *Notch* (frequently mutated in human SCC, [33, 34]). First, tumors were stained using an antibody against cleaved Notch1, i.e. recognizing the Notch 1 intracellular domain (Nicd) which relocates to the nucleus up on activation. Nicd expression is affected by mutations in *Notch1* [33]. Our staining (method given in Supplementary Information) showed highly variable Nicd expression in the tumors, and was not conclusive on possible mutations. Next, we sequenced 10 tumors (6 from the sub-sunburn group, 4 from the sunburn group) for mutations in *Notch1* and *Notch2* (focusing on domains most often affected in human cutaneous SCCs [33, 34]; see Supplementary Information). None were found.

## DISCUSSION

In this study we showed that there is a difference in tumor initiation by a single UV overexposure compared to the same total dose delivered in very small sub-sunburn dose fractions: only in the latter case tumors grew persistently after 20 weeks of TPA treatment. In absence of epidermal hyperplasia during exposure, both UV regimens should have produced the same initial DNA damage (CPDs), but spreading this damage over small daily aliquots was apparently linked to the ultimate yield of persistent (in situ) SCCs. In contrast to the single sunburn UV exposure, the fractionated sub-sunburn UV dose resulted in CPD-retaining skin cells which persisted over a week after the last UV exposure. These stationary, non-dividing cells lagging behind in repair of CPDs had apparently accumulated part of the undetectably small daily aliquots of CPDs. Besides fibroblasts in the upper dermis, sparse basal cells in the epidermis showed retention of CPDs; in an earlier study we demonstrated the latter cells (CPD-retaining basal cells, CRBCs) to be quiescent stem cells (QSCs) [26]. TPA induced division of these CPD-retaining epidermal cells, but evoked no discernable response in the CPD-retaining fibroblasts (see Figure 1 and our earlier study [27]). Forcing the CRBCs with their high CPD loads to proliferate is likely to increase the risk of mutations and thus have a special bearing on epidermal UV carcinogenesis. In a natural situation the forced proliferation may be evoked by an irritant or a mild sunburn inducing (transient) hyperplasia [25].

With regard to AK/SCC, our data show that a severe sunburn from a single overdose appears to be less hazardous than spreading the same dose over multiple small exposures. This finding appears to be in line with the older finding that single weekly exposures are less effective in inducing SCCs than daily doses mounting up to the same weekly dose [35]. This could be related to the extent to which quiescent stem cells are activated in the hyperplastic response and the speed at which CPDs are removed in the epidermal turnover.

The daily UV dose fractions in our experiment were low enough not to induce any proliferation of quiescent stem cells and hyperplasia, as can be seen in Figure 1A. The undetectably small aliquots of CPDs induced daily were being lost mostly in epidermal turnover. This DNA damage only accumulated in long residing (quiescent stem) cells if the repair lagged behind – which it apparently did for CPDs but not for 6-4 photoproducts [26]. In literature there are several markers described for the long-residing non-dividing QSCs, e.g. Wif-1 [36], Lrig1 [37] and Dll1 [38]. We checked for double staining with anti-CPD to see if they would co-localize (see Supplementary Table S2 on antibodies used). However, these markers either did not label interfollicular basal cells at all in mice (Lrig1+, Dll1) or did not double stain (Wif1, which was predominantly expressed suprabasally in TPA driven hyperplasia; data not shown). Thus, we could not identify a specific phenotypic marker of these cells which could be exploited to eliminate these cells specifically (e.g. by induction of a transgene for diphtheria toxin). Such an eliminatory intervention could serve to proof the suspected causal role of CPD-loaded QSCs in raising persistent SCCs.

The retention of UV-induced DNA damage in quiescent stem cells is reminiscent of the retention of chemical DNA adducts in quiescent cells as the tumor-initiating step in a two-stage regimen of chemical skin carcinogenesis (i.e., tumor initiation by a genotoxic agent followed by “tumor promotion” by repeated applications of a cell proliferation inducing agent, such as TPA) [39]. This regimen gives rise to PCR-detectable oncogenic *H-ras* mutations one week after tumor initiation, months before the occurrence of skin tumors carrying such mutations [40].

Only in the sub-sunburn group, corresponding with induction of CPD-retaining cells, we found clear, but rare, p53-overexpressing and p53-mutant clusters of cells (“p53 patches”) long before onset of tumors, albeit in very low numbers. This is somewhat in contrast with earlier results [27] where no p53-mutant patches could be detected, but high numbers of small wild-type p53 expressing patches were found, speculated to reflect a reaction of TPA-driven cells to proliferative stress from DNA damage. This discrepancy is most likely attributable to a difference in techniques (detection in epidermal sheets or cross sections) and sensitivity (difference in antigen retrieval), despite using the same antibodies.

Only in the sub-sunburn group we found CRBCs and p53 patches. As the regimen of 1/7 MED/day for 40 days is unlikely to induce p53-mutant patches [17], this suggests that some CRBCs attained *p53* mutations after TPA-induced proliferation and grew out to p53 patches.

But the p53 patches were very sparse (3.8/cm^2^ on average), and none of the ensuing persistent (in situ) skin carcinomas carried any *p53* mutations. Very much in contrast to what we found earlier with life-long chronic exposure of SKH1 hairless mice which gave rise to multitudes of p53 patches and subsequently carcinomas with abundant *p53* mutations with the “UV signature” (>70% of tumors) [13], also at low daily doses [18]. This lack of *p53* mutations could conceivably hamper progression to malignancy (high percentage of in situ SCCs/Bowenoid tumors and low percentage of SCCs in present experiment in comparison to earlier experiments with chronic, life-long daily UV exposure, Table 1) [30].

Of human SCCs, 50 – 70 % have been reported to carry UV-related *P53* mutations [10, 41, 42], and similar percentages in AKs [41]. But in some studies much lower percentages have been reported in SCCs (e.g. 15% [43]) and in “low grade” AKs (7% [44]). *NOTCH* genes proved to be even more abundantly UV-mutated than *P53* [33, 34]. The lack of *p53* mutations in the current experiment is evidently due to early termination of the UV regimen, which might also explain the lack of mutations in *Notch*. This apparently also resulted in only a few *p53* mutant clones (p53 patches). Considering the overall high percentages of human SCCs with UV signature mutations in *P53* and *NOTCH*, one is led to infer that most of these tumors are induced by life-long, or at least decades of, chronic UV exposure. Even in temperate climate with low ambient UV, high frequencies of UV-related *P53* and *NOTCH* mutations were found in sun-exposed adult skin [45, 46].

We have earlier shown that in contrast to chronic UVB exposure, chronic UVA exposure of SKH1 hairless mice induced SCCs of which a large majority (85%) lacked *p53* mutations [47]. Like low level UVB, UVA exposure appears to be much less effective in stimulating cell proliferation and hyperplasia than higher level UVB [48], and it may therefore also be targeted more at QSCs and deeper residing fibroblasts.

The AK/SCCs initiated by low level daily UVB exposure (40 days) – associated with CPD-retaining skin cells – and promoted to grow out by repeated applications of TPA – specifically stimulating CRBCs - differ from earlier SCCs induced by life-long chronic UVB exposure in that they harbor no *p53* and *Notch* mutations, and they differ from chemically initiated and TPA-promoted tumors in that they harbor virtually no oncogenic (*H-*)*Ras* mutations. Thus, there appears to be another as yet unknown tumor initiator/driver which may also be involved in the earliest stages of skin carcinogenesis by life-long chronic UV exposure.

Histopathology (Table 1) was mainly based on tumor samples from mice sacrificed at the end of the experiment and from a few mice sacrificed earlier. Hence, most of the samples were from persistently growing tumors from the sub-sunburn group, mostly (in situ) SCCs and precursor AKs. As most of the tumors regressed in the sunburn group, few tumors could be analyzed of which most from a mouse sacrificed early, 5 out of 7 AKs (see footnote Table 1). The low percentage of SCCs and high percentage of Bowenoid tumors (in situ SCCs) in the sub-sunburn group in comparison with earlier experiments with chronic exposures (Table 1) suggests that tumor progression stagnated in the ‘in situ’ stage without further UV exposure, despite TPA-driven outgrowth of the tumor. As virtually all of the suspected AK/SCCs, i.e., endophytically growing tumors, regressed upon discontinuation of the TPA regimen in the sunburn group, we have very little information on the histopathology of these regressing tumors. AKs are, however, known to regress (e.g. if no longer subjected to sun or UV exposure) [49, 50]. The AK/SCC-like tumors ≥ 3 mm that regressed are not likely to have been truly malignant SCCs. Besides AKs, they may also have been benign keratoacanthomas which are hard to distinguish from SCCs but, unlike SCCs, are known to regress spontaneously in man [51].

Acute overexposure and especially chronic exposure to (solar) UV radiation are associated with the development of squamous cell carcinomas. The present study shows that a single sunburn dose differs in initiating epidermal tumors from an equal dose fractionated into sub-sunburn exposures: the latter results in non-regressing tumors whereas the tumors from the single exposure went into regression upon cessation of tumor promotion by TPA treatment. Interestingly, the initiation of non-regressing tumors was associated with DNA damage-retaining QSCs in the epidermis which were forced to divide by TPA treatment. Surprisingly, none of the persistent skin tumors showed mutations in the common driver genes, i.e. no mutations in *p53*, *Notch* and *Ras*. We infer that none of these common driver mutations were involved in the UV-driven tumor initiation. Although the as yet unknown tumor-initiating mutations may have been forced to occur by TPA-induced proliferation of DNA damage-retaining cells, they may also constitute the earliest tumor initiation events in skin carcinogenesis by life-long chronic UV exposure.

## MATERIALS AND METHODS

### Mice

As legally required, the experiments were performed with the approval of the Leiden University Medical Centers' ethics committee for animal experiments (DEC10229) and executed according to EU regulations on animal experiments (Directive 2010/63/EU).

Male and female albino Crl: SKH1-HR hairless mice (breeding pairs from Charles River, Sulzfeld, Germany) entered the experiment at 6-9 weeks of age. The mice were kept individually in Macrolon type 1 cages with standard chow and tap water *ad libitum*, in a room at 25 ± 2°C, 50% humidity and illuminated by yellow fluorescent tubes (no UV radiation) in a 12 h-on-12 h-off cycle.

### UV radiation

For the fractionated UV dose we used the protocol used previously by our group [26, 27]; this regimen yielded CPD-retaining skin cells. In short, Philips TL-12/40W tubes were used (output of 54% in UV-B, 280-315 nm, and 46% in UV-A, 315-400 nm). The minimal erythemal/edemal dose (MED) was determined to be 500 J/m^2^ UV for SKH-1 hairless mice under these lamps. The sub-sunburn group (n=20) received 40 days 70 J/m^2^ UV a day which is equal to 0.14 MED/day. The sunburn group (n=21) received the same dose unfractionated in a single exposure: 2.8 kJ/m^2^ = 5.6 MED at day 1, a just tolerable dose without wounding the skin. Based on an extrapolation by using an earlier quantitative dose-response model of ours [52] we calculated that virtually no tumors are to be expected from such an initial total dose alone (0.006 tumors > 2 mm per mouse after 2 years; this model makes no distinction between delivering the dose in a single day or 40 days). In earlier studies it was indeed found that a single high UV exposure alone did not give rise to tumors [29] unless the exposure was high enough to cause ulcerations where a small number of benign papillomas occurred preferentially at the edges of scars and a few rare carcinomas after the most extreme doses [53].

The UV overdose causes apoptosis in the basal layer [54], transient strong epidermal hyperplasia with increased proliferation in the basal layer, which makes it impossible for CPD-retaining epidermal cells to persist [25, 27]. Additional mice in each group (n=4 at each time point) were used to assess CPDs and p53 mutant foci in the course of the TPA treatment (see below). See for a schematic overview of the experimental outline including the time points of UV exposure and of taking samples Supplementary Figure S1.

### TPA application

From day 47 onward both groups of mice received TPA treatment twice a week for a period of 20 weeks to promote tumor development; the TPA also induced proliferation of quiescent stem cells. TPA (12-O-tetradecanoylphorbol-13-acetate, Sigma-Aldrich, Zwijndrecht, The Netherlands) was dissolved in acetone to a concentration of 100 μg/ml. Each time, about 100 μl TPA was applied to an area of dorsal skin of approximately 6 cm^2^ using a fine brush. By 20 weeks of TPA treatment some mice had started to scratch and wound themselves. At that point, TPA treatment was discontinued for all mice. Scratching had occurred equally in both groups affecting a minor dorsal area (<10%) of skin in about 15% of the mice.

### Tumor assessment and pathology

For the tumor assessment, mice were inspected every other week. Tumors were scored on maps to record location, size and tumor type. The first tumor was scored when present for at least two successive inspections. The experiment was terminated after 280 days, when most mice in the sub-sunburn group developed a substantial tumor load. Only mice free of tumors (>4 mm) were maintained up to 350 days to complete Kaplan-Meier plots of tumor-free survival.

With macroscopic tumor assessment two types of tumors were distinguished: papillomas (exophytically growing tumors, protruding from the skin, often pedunculated) and “non-papilloma” tumors (endophytically growing tumors, gradually sloping at the edges) [31]. A small number of tumors arising in scratch-scarred skin was not taken into account and censored. Mice were taken out of the experiment either when in poor health (very rarely) or their tumor load was getting too high (> 0.5 cm^3^; also rarely) or at the end of the experiment. Tumors were isolated from the sacrificed animals as described below. At the end of the experiment tumors ≥ 3 mm across were renumbered and a random selection without further information was submitted for pathology.

### Tissue preparation

Mice were sacrificed using CO_2_ asphyxiation. For anti-CPD and anti-p53 immunohistochemical stainings dorsal skin was obtained using a template. One piece of 5×16 mm was directly frozen in liquid nitrogen, another piece of 5×16 mm was fixed overnight in PBS-buffered 4% formaldehyde solution (Addedpharma, Oss, The Netherlands) and embedded in paraffin. Furthermore two skin samples of 7.5×32 mm were obtained to prepare epidermal sheets. To separate the dermis from the epidermis, one sample was treated with 100 μg/ml thermolysin (Sigma-Aldrich) in PBS containing 1 mM CaCl_2_ (pH 7.8) and incubated overnight at 4°C. The other sample was treated with 20 mM EDTA (Baker, Deventer, The Netherlands) in PBS for 2 hrs at 37°C. After removal of the dermis, epidermal sheets were stored in 70% alcohol until further use. Tumors ≥3 mm were isolated and one half was frozen in liquid nitrogen and the other half was fixed in PBS-buffered 4% formaldehyde solution and embedded in paraffin.

### Immunohistochemistry

For anti-CPD staining frozen tissue was embedded in Tissue-tek (Sakura Finetek Europe, Zoeterwoude, The Netherlands) and 6 μm thick cryosections were cut. The sections were fixed in acetone for 10 min, and boiled for 5 min in 10 mM citrate buffer (pH 6.0) for antigen retrieval. The sections were slowly cooled down to 37°C and blocked for 20 min with MilliQ water containing 50% methanol and 0.3% H_2_O_2_. Next, the samples were pre-incubated with 10% normal goat serum (NGS) (Dakocytomation, Heverlee, Belgium) in PBS with 1% BSA for 20 min followed by overnight incubation at 4°C with anti-cyclobutane pyrimidine dimer antibody (1:1000, Kamiya, Seattle, USA) in PBS containing 1% NGS and 1% BSA. Goat anti-mouse (IgG_1_)-biotin (Southern Biotechnologies, Birmingham, USA) 1:200 in PBS 1%BSA was incubated 1hr at RT, followed by incubation with avidin-biotin peroxidase complex (Vectastain Elite, Burlingame, USA) for 45 min. AEC (3-Amino-9-ethylcarbazole Sigma-Aldrich) was used to make the CPDs visible. The sections were counterstained with hematoxyline (Klinipath, Duiven, The Netherlands) and mounted in Kaisers' Glycerin.

For p53 patch staining, epidermal sheets obtained using EDTA or thermolysin were used and stained as described previously [17, 55] with some minor modifications (see Supplementary Information).

The protocols for the anti-CM5 staining and the staining for Notch 1 intracellular domain (Nicd) on tumors are also described in the Supplementary Information.

Images were acquired with a Zeiss Axioplan 2 microscope using the 10x and 20x objectives, Axiocam camera and dedicated software. Final pictures were formatted in Adobe Photoshop CS6 or Adobe Illustrator CS6.

### PCR and mutation analyses of tumors (p53, Ras and Notch)

Frozen tumors were incubated in RNAlater-ICE (Life Technologies Europe BV, Bleiswijk, The Netherlands) overnight at −20°C; tumor material was isolated the next day and used for DNA isolation. DNA was isolated using the DNeasy Blood and Tissue kit (Qiagen Benelux BV, Venlo, The Netherlands) according to manufactures protocol including RNase A treatment (Qiagen Benelux BV). The DNA concentration was measured using a Nanodrop and about 18 ng was used as template for PCR. The PCR was performed using the Platinum Taq DNA Polymerase kit (Life Technologies Europe BV) in a Bio-Rad C1000 touch thermo cycler (30 cycles three step protocol for *p53* and a touchdown PCR for *Ras* and *Notch*). Conserved domains in *p53*, exon 4-8 were inspected for mutations. Stretches around codons 12 and 61 in *H-, K-* and *N- Ras* were sequenced to check for oncogenic mutations. And for *Notch1* and *Notch2* we looked at the mouse equivalents of mutated exons in human [33, 34] (see also Supplementary Information).

In Supplementary Information Table S1 the sequences and annealing temperature of the primers for the different PCRs are given. PCR products were isolated using the Nucleospin Gel and PCR clean-up kit (Machery-Nagel, via Bioke, Leiden, The Netherlands). Products were either isolated directly from the PCR mixture or from a 1% agarose gel according to manufactures protocol (for *Notch* the isolation was done slightly differently, see Supplementary Information). The PCR products were sequenced in both directions using forward and reverse PCR primers in combination with Sanger sequencing (Macrogen, Amsterdam, The Netherlands). Sequences were analyzed using Mutation Surveyor V4.0.9 software (Softgenetics LCC, State College, USA) and sequences were double checked by visual inspection for mutations that the program might have missed. Mutations were scored when present in both the forward and reverse sequence, and when the wild type signal was correspondingly reduced.

### Data processing and statistical analyses

Tumor development was scored in two ways: a) tumor-free survival (Kaplan-Meier plots) and b) tumor yield (average number of tumors per survivor). Statistical significant differences in tumor-free survival were determined by the Mantel-Cox test (in Graphpad Prism 6). Differences in tumor yields were analyzed with univariate two way ANOVA (testing for group and gender differences) and with a t-test for independent samples, not assuming equal variances for final differences between groups (in IBM SPSS Statistics 20). Statistical significance was set at p≤0.05. Means were given with standard error of the mean (SEM) unless otherwise stated. Graphs were generated in Graphpad Prism 6 and formatted in Adobe Illustrator CS6.

## SUPPLEMENTARY INFORMATION FIGURES AND TABLES


