# Supplementary material for: Fractionation of a tumor-initiating UV dose introduces DNA damage-retaining cells in hairless mouse skin and renders subsequent TPA-promoted tumors non-regressing
Source: Oncotarget. 2016 Jan 18;7(7):8067–77. doi: 10.18632/oncotarget.6932 (PMC4884976; doi:10.18632/oncotarget.6932)
Supplement: Supplementary file 1 [file oncotarget-07-8067-s001.pdf]

SUPPLEMENTARY INFORMATION

METHODS

P53 patch staining on epidermal sheets

Thermolysin sheets (see materials and method) were fixed with PBS-buffered 4% formaldehyde solution for 10 min and EDTA sheets were fixed for 20 min in aceton. For both antigen retrieval was performed by boiling 5 min at 110 °C in 10 mM citrate buffer (pH 6.0). Endogenous peroxidases were blocked by incubating the sheets in methanol containing 1.5% H_2O_2 for 20 min. The sheets were pre-incubated for 10 min with 5% normal serum/0.2%BSA/0.1% saponine (Sigma-Aldrich) in PBS; goat serum for CM5 and rabbit serum (Dakocytomation) for Pab240 staining. PBS containing 0.5% BSA and 0.1% saponine was used to dilute the primary antibodies, either Pab240 (mutant specific, mouse monoclonal anti-p53 antibody, Neomarkers, Duiven, the Netherlands) 1:25 for thermolysin sheets or CM5 (rabbit polyclonal anti-p53 antibody, Monosan (Sanbio), Uden, The Netherlands) 1:250 for EDTA sheets. The primary antibodies were incubated overnight at 4°C. Secondary antibodies, rabbit anti mouse (IgG_1)-biotin (Southern Biotechnologies, Birmingham, USA) 1:200 and goat anti rabbit (IgG)-biotin (Vector Laboratories, Peterborough, UK) 1:300 diluted in PBS/BSA/saponine were incubated for 45 min. Sheets were subsequently incubated with avidin-biotin peroxidase complex (Vectastain Elite, Burlingame, USA) for 45 min and stained with 3,3′-diaminobenzidine (DAB, Sigma-Aldrich). Sheets were mounted with the basal side up in Kaiser’s glycerin.

Anti-CM5 and Notch staining on tumors

Anti-CM5 and Notch staining on tumors was performed on paraffin embedded samples that were sectioned at 5 µm thickness. For both stainings, antigen retrieval was performed by boiling the samples 5 min at 110°C in 10 mM citrate buffer (pH 6.0). For the anti-CM5 staining the samples were incubated with blocking solution (methanol containing 1.5% H_2O_2) and subsequently with anti-p53 (1:2000, CM-5, Monosan (Sanbio), PSX1021) in 5% NGS in PBS/1% BSA overnight at 4°C. The secondary antibody, goat-anti-rabbit-IgG-biotin (1:300, Vector, BA1000) in PBS/1%BSA was incubated for 1 hr at RT. The avidin-biotin peroxidase complex combined with DAB was used for visualization in the same way as for the p53 patch staining. Samples were embedded in Kaisers Glycerin.

For the Notch staining samples were incubated 10 min with avidin block and 10 min with biotin block (Biotin blocking system X0590, Dako cytomation) and blocked with 2% NGS, in PBS/1% BSA. Subsequently, they were incubated with anti-cleaved Notch1 antibody (Val1744, D3B8, Rabbit mAb, Cell signaling) overnight at 4°C. Goat-anti-Rabbit-IgG-biotin (1:300) was incubated for 1 hr at RT, followed by 1 hr incubation with streptavidin-biotinylated horseradish peroxidase complex (1:100, RPN1051V, Amersham) at RT. DAB was used for visualization (3,3′ Diaminobenzidine (D5905, Sigma) and samples were embedded in Depex.

Bead purification of *Notch* pcr products

Pcr products were purified using AMPure XP beads (A63881, Beckman-Coulter) using a protocol similar to that of the manufacturer. In short 25 µl H_2O and 50 µl of AMPure XP beads was added to 25 µl of pcr product, mixed by pipetting and incubated for 5–8 min at RT. The samples were placed on a Dynal MPC®-96S magnet (120–27, Invitrogen), and the supernatant was removed. The samples were washed twice with 200 µl 80% ethanol, the supernatant was removed and the pellets were dried for approximately 2 min in a heath block at 37°C. 15 µl of 10 mM Tris-HCl buffer pH 7.5 (diluted from 1M Tris-HCl buffer, 15567–027, Life technologies) was added to the samples; samples were vortexed until all samples were homogenous and incubated for 30s-2min at RT. The plate was placed on the magnet again to separate the beads from the solution and the eluate was transferred to a new plate.

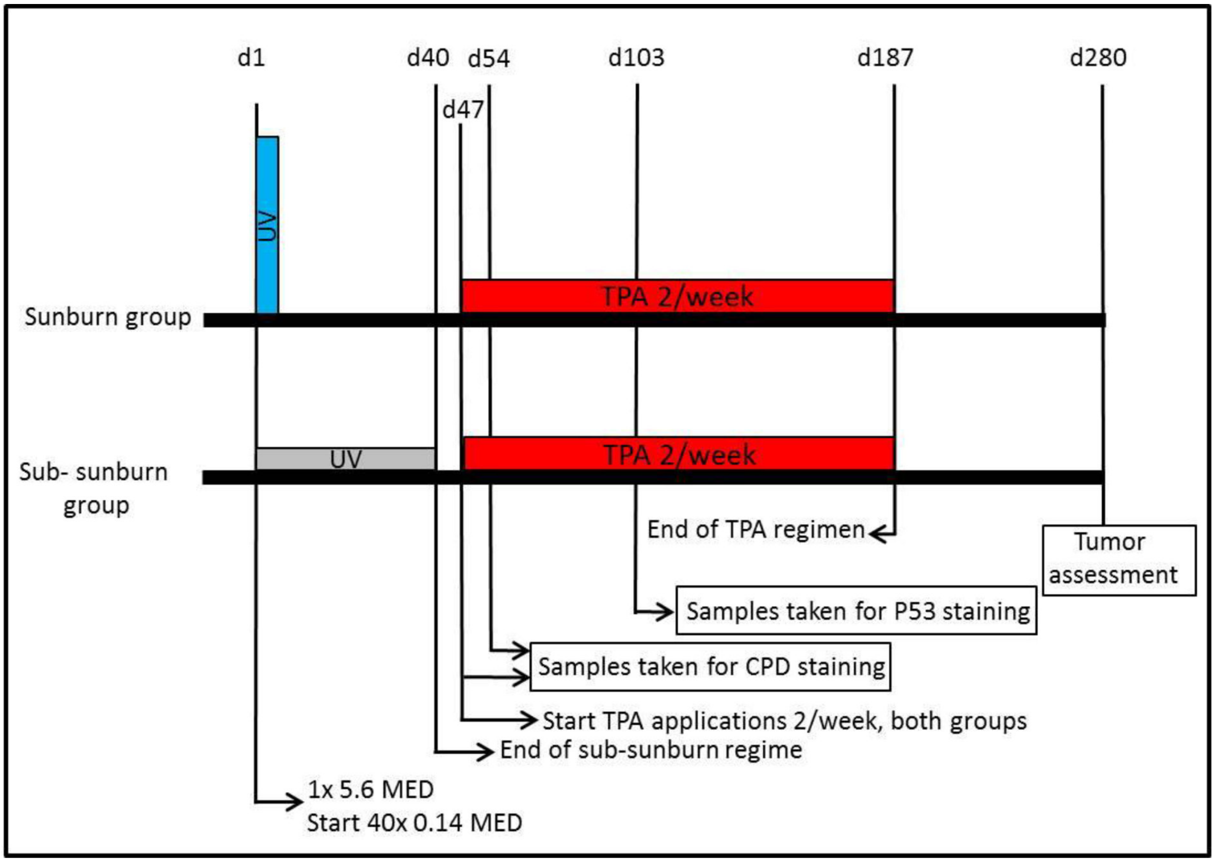

Supplementary Figure S1: Experimental outline. Time line of the experiments including time points of sample collection for analyses, d= day. The sunburn group received their single UV overexposure (5.6 MED) on day 1 instead of day 40 to avoid interference of the UV induced hyperplasia with hyperplasia from TPA, and to maximize the time for possible fixation of mutations.

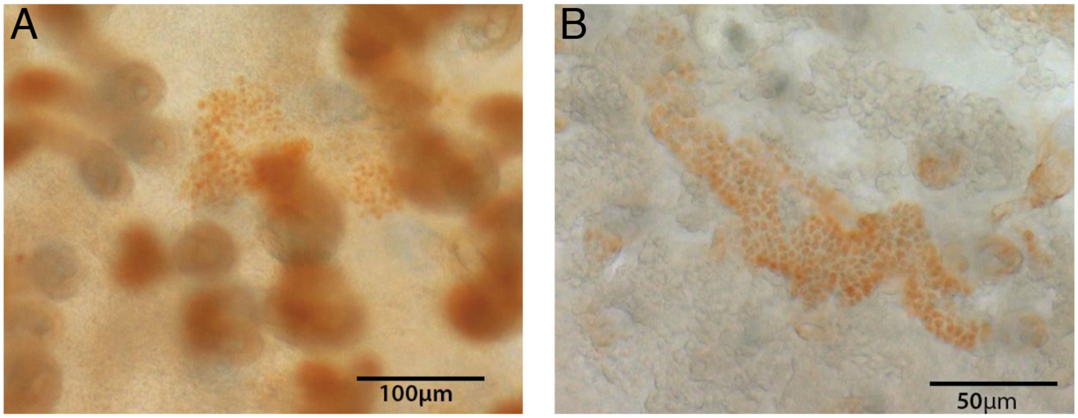

Supplementary Figure S2: P53 patches developed after 40 days subsunburn UV and 8 weeks TPA applications. Patch detected using the CM-5 antibody **A.** or the Pab240 antibody **B.**

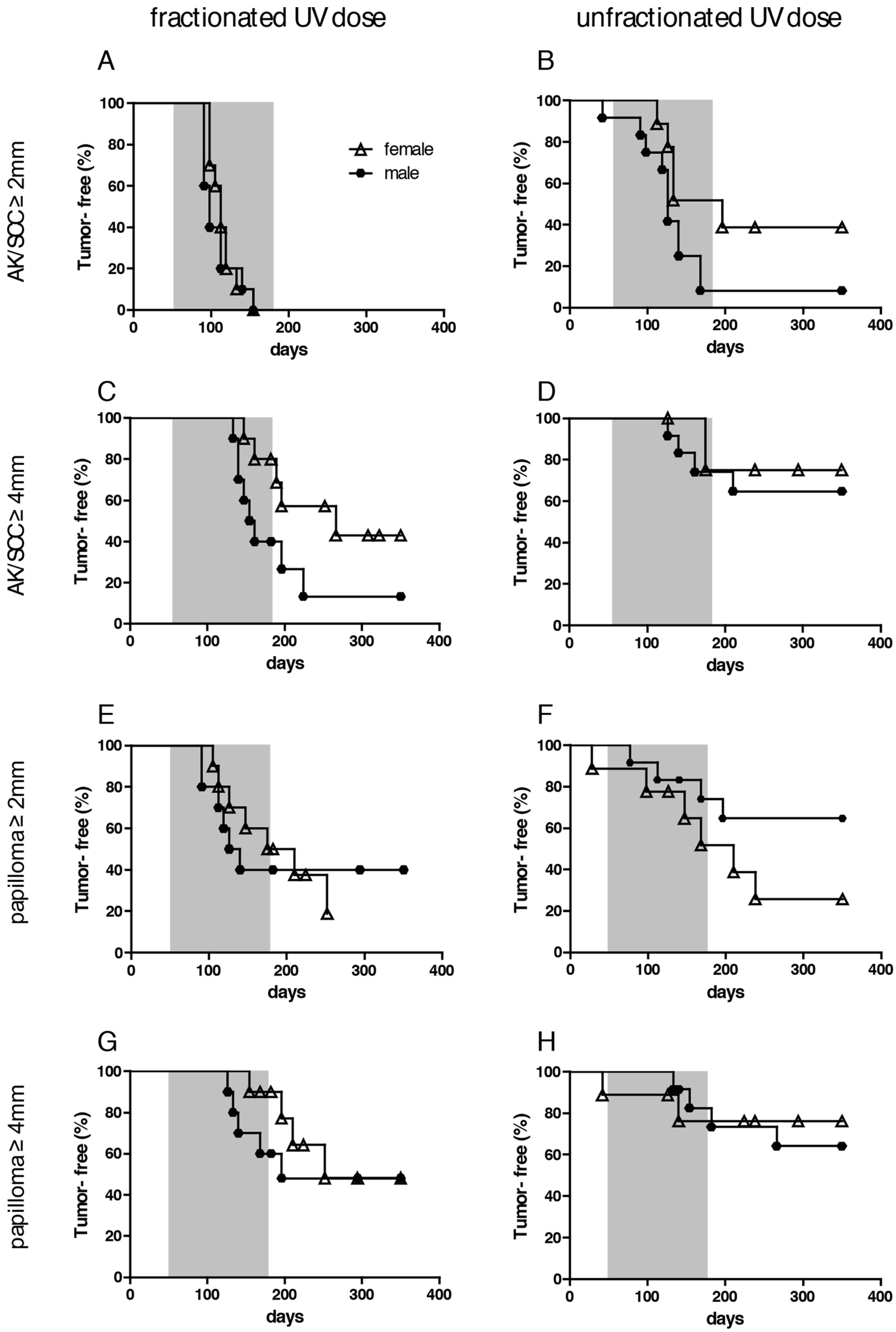

Supplementary Figure S3: Gender differences in tumor-free survival.(Δ) female and (•) male**.** Result for fractionated UV in left column and unfractionated in right column, and for AK/SCC in top 4 panels and for papillomas in bottom 4 panels; for tumor sizes > 2mm in **A,B,E,F** and > 4mm in **C,D,G,H**. The grey area in the graphs represent the period in which TPA was applied. Only AK/SCC ≥ 4 mm fractionated dose **C.** shows borderline gender difference ($p = 0.06$).

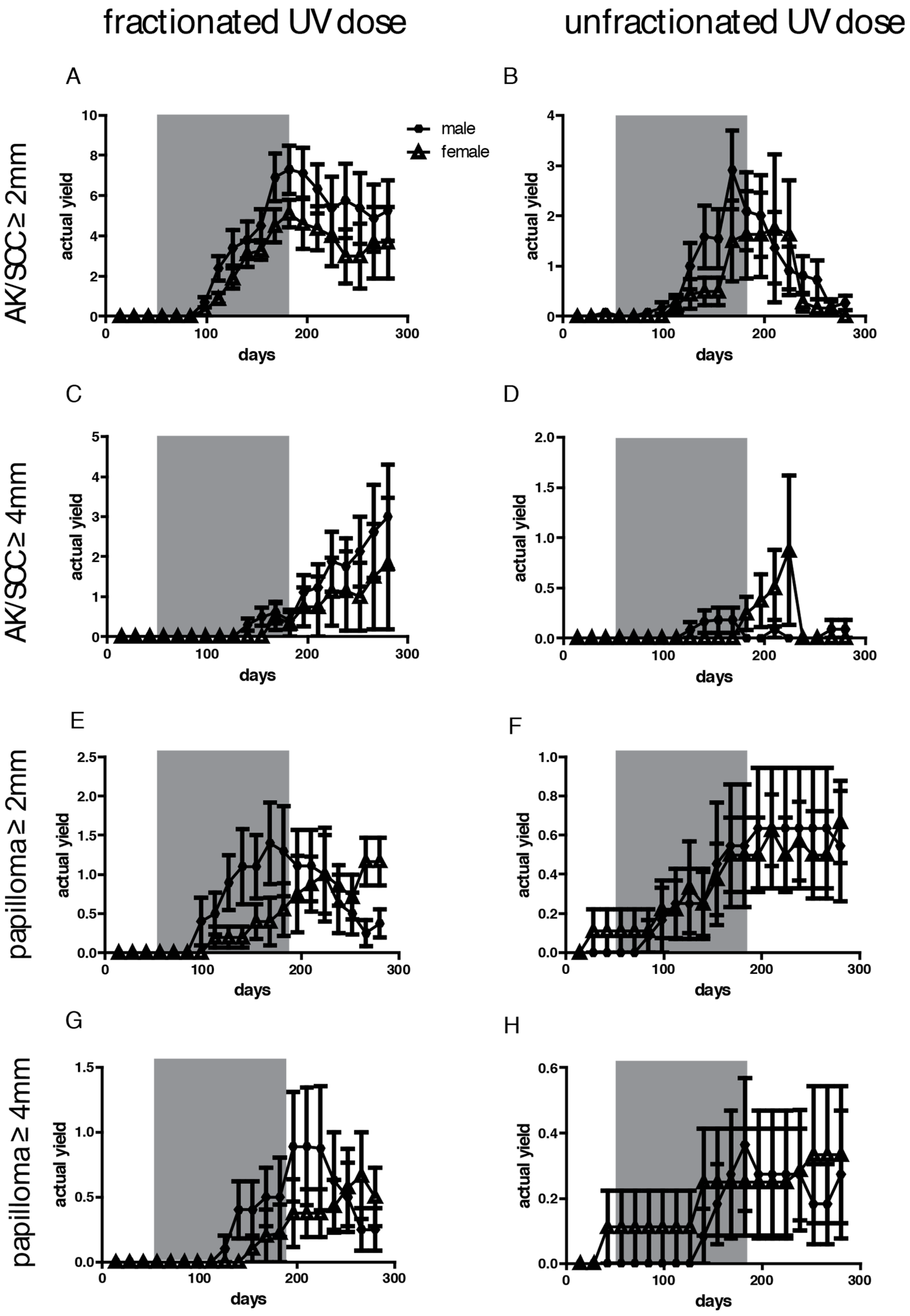

Supplementary Figure S4: Gender differences in tumor yields. (Δ) female and (•) male. Result for fractionated UV in left column and unfractionated in right column, and for AK/SCC in top 4 panels and for papillomas in bottom 4 panels; for tumor sizes > 2mm in **A,B,E,F** and > 4mm in **C,D,G,H**. The grey area in the graphs represent the period in which TPA was applied. Error bars show SEMs; no significant differences.

Supplementary Table S1: Primer sequences and PCR protocols for amplification of p53, Ras and Notch DNA

	Forward primer	Reverse primer	PCR protocol
P53 exon 4	cccctgtcatcttttgtccc	tcagggcaaaactaaactct	AT 55°C; 32 cycles
P53 exon 5	agttccccaccttgacacct	agagcaagaataagtcagaa	AT 55°C; 32 cycles
P53 exon 6	actggcagcctcccatctccc	gtcaactgtctctaagacgca	AT 55°C; 32 cycles
P53 exon 7	gtagggagcgacttcacctg	cagaagctggggaagaaaca	AT 55°C; 32 cycles
P53 exon 8	cttgtgctggtccttttcttg	aggagagagcaagaggtgact	AT 55°C; 32 cycles
Hras codon 12	agtgtgcttctcattggcag	gcaggtaggcagagctcacc	TD 65°C > 58°C +30x AT 60°C
Hras codon 61	cccactaagccgtgttgttt	tggtgttgttgatggcaaat	TD 57°C > 50°C +30x AT 52°C
Kras codon 12	tgtaaggcctgctgaaaatg	gcacgcagactgtagagcag	TD 65°C > 58°C +30x AT 60°C
Kras codon 61	ccagactgtgtttctccctt	tgccaactttcttattcaac	TD 57°C > 50°C +30x AT 52°C
Nras codon 12	gactgagtacaaactggtgg	ctctatggtgggatcatatt	TD 65°C > 58°C +30x AT 60°C
Nras codon 61	ggtgagacctgcctgctgga	atacacagaggaacccttcg	TD 65°C > 58°C +30x AT 60°C
Notch1 Exon 2	gctttaatctccgtccacca	gggctctgctctgacaaaag	TD 65°C > 58°C +30x AT 60°C
Notch1 Exon 3	ctgtggccatgagagatgag	ctaagggtcccaaccgtga	TD 65°C > 58°C +30x AT 60°C
Notch1 Exon 4	aaatctgccctgggttcac	ggacatggacaaccaggcta	TD 65°C > 58°C +30x AT 60°C
Notch1 Exon 5	tgtccagagcagaacattgc	gcggtgtgcagttttgttag	TD 65°C > 58°C +30x AT 60°C
Notch1 Exon 6	cagagcagggagctcatctt	gtggtgagccaaccaagttt	TD 65°C > 58°C +30x AT 60°C
Notch1 Exon 9	cccgtatgagggagggttat	cctcaaaggagtctgggaag	TD 65°C > 58°C +30x AT 60°C
Notch1 Exon 12	agtgggtggaaaagggtgag	tgacacccagcttgtgacat	TD 65°C > 58°C +30x AT 60°C
Notch1 Exon 13	gctctaggaggggtaggctct	gcccaaggctacagctagtc	TD 65°C > 58°C +30x AT 60°C
Notch1 Exon 16	ccaggctcactgggttctat	ctgacttcccatccaccatc	TD 65°C > 58°C +30x AT 60°C
Notch1 Exon 17	aggggagaagcactggagat	aggcaccgaaacataggatg	TD 65°C > 58°C +30x AT 60°C
Notch1 Exon 19	ccaggtaggcatggagttgt	ctggccctctgaaggtagtg	TD 65°C > 58°C +30x AT 60°C
Notch1 Exon 21	atgggcagtagtcccgagag	gcaacactcacgctgatacc	TD 65°C > 58°C +30x AT 60°C

(*Continued*)

	Forward primer	Reverse primer	PCR protocol
Notch1 Exon 25	ACCTTTCACAGGCCACAGAC	ctgagccctgggactctaaa	TD 65°C > 58°C +30x AT 60°C
Notch1 Exon 26	atgtctatccctgcccattg	cctccagggttaagctggtt	TD 65°C > 58°C +30x AT 60°C
Notch1 Exon 27	gggagtcagagctggtgtgt	gggatttgaacccttgtcct	TD 65°C > 58°C +30x AT 60°C
Notch1 Exon 30	ccaagtgatgaggctctgct	gggaatgtgtgcatgtgtct	TD 65°C > 58°C +30x AT 60°C
Notch1 Exon 31	ccagggctatggttgaatgt	caaaatgcagtgtggaggtg	TD 65°C > 58°C +30x AT 60°C
Notch1 Exon 34.1	gcagcacctgcttcccta	ctgccatctcaggcttgg	TD 65°C > 58°C +30x AT 60°C
Notch1 Exon 34.2	agtcaccccatggctacttg	cggttgtacatctgcctgac	TD 65°C > 58°C +30x AT 60°C
Notch1 Exon 34.3	ccttgtccccgattatttacc	aaaaatggaaattcttgtcctct	TD 65°C > 58°C +30x AT 60°C
Notch1 Exon 7–8	gaaaagcagcacccactcac	ccctgctgcctgctcttag	TD 65°C > 58°C +30x AT 60°C
Notch1 Exon 10–11	agcaaccaaggggttaggat	gcatgacatgtggtcagctt	TD 65°C > 58°C +30x AT 60°C
Notch1 Exon 14–15	atgtggccttgtcctcagac	tcagcgaggaacagaacctt	TD 65°C > 58°C +30x AT 60°C
Notch1 Exon 23–24	aggacagagcacagtcagca	aagagtcggtcagggttcag	TD 65°C > 58°C +30x AT 60°C
Notch1 Exon 28–29	gatggaggagagtgggtgag	agcagagcctcatcacttgg	TD 65°C > 58°C +30x AT 60°C
Notch1 Exon 32–33	caggcttgcatttagatcacc	tgctgttgtcccagacactc	TD 65°C > 58°C +30x AT 60°C
Notch2 Exon 2	gtgcaggggtgctaagaaag	gccagcaacacttcacagaa	TD 65°C > 58°C +30x AT 60°C
Notch2 Exon 3	catcgttttctggtctttgga	tgcacagtacaggtcaccac	TD 65°C > 58°C +30x AT 60°C
Notch2 Exon 4	gacagagctgctgagtgtgg	tctgggaagtacaccctcca	TD 65°C > 58°C +30x AT 60°C
Notch2 Exon 5	tgctgggatatgcactgaaa	cccactttgctaagagtttcg	TD 65°C > 58°C +30x AT 60°C
Notch2 Exon 6	gctgttagggtgccagtcat	ctgcattccagaatggtcct	TD 65°C > 58°C +30x AT 60°C
Notch2 Exon 7	tggttctgcttttgacaagaaa	actttctgagccgacacgac	TD 65°C > 58°C +30x AT 60°C
Notch2 Exon 8	tgtctttgtgggaactctgg	tcagtgcatggagtcagtgag	TD 65°C > 58°C +30x AT 60°C

(*Continued*)

	Forward primer	Reverse primer	PCR protocol
Notch2 Exon 9	tgcacatttttgcttccttg	gggttttggagagcacagg	TD 65°C > 58°C +30x AT 60°C
Notch2 Exon 10	accccttggcatacactctg	cagctaggggaagacagtcg	TD 65°C > 58°C +30x AT 60°C
Notch2 Exon 11	ggtgtagactcagcgtgctc	caccagacacagagcaggaa	TD 65°C > 58°C +30x AT 60°C
Notch2 Exon 12	aagtgctgacattcctgtgg	gagcagactgcaaaccttcc	TD 65°C > 58°C +30x AT 60°C
Notch2 Exon 13	taaccccgtcccttgagaat	ccaactgggccaatcagtaa	TD 65°C > 58°C +30x AT 60°C
Notch2 Exon 14	ggaaaggtgaaactggcttt	gggtgtaccacaagggaaga	TD 65°C > 58°C +30x AT 60°C
Notch2 Exon 15	caccaggagagctggtgagt	cttggagaccacgagtgaca	TD 65°C > 58°C +30x AT 60°C
Notch2 Exon 16	agtcttgggacgccatcttt	ttccagctagtcgctcccta	TD 65°C > 58°C +30x AT 60°C
Notch2 Exon 18	gggtcatctgccttcacagt	ggttcatccttgccctacaa	TD 65°C > 58°C +30x AT 60°C
Notch2 Exon 19	cagggcctcagctcacac	ctactgtggggagccaggt	TD 65°C > 58°C +30x AT 60°C
Notch2 Exon 22	gcttagtgggatggaagcaa	tagagggaatgtccctggtg	TD 65°C > 58°C +30x AT 60°C
Notch2 Exon 23	cgggttcagtggatttttgt	cctaagctggtcttggcttg	TD 65°C > 58°C +30x AT 60°C
Notch2 Exon 24	tctgaggctgcttcctatcc	ggctcaatctcagctcccta	TD 65°C > 58°C +30x AT 60°C
Notch2 Exon 25	gccctcacctgttgactgat	ccgagcagttaggagcaaga	TD 65°C > 58°C +30x AT 60°C
Notch2 Exon 26	gaggctgcagtgtgttctga	tcaaagtgcccagctgtatg	TD 65°C > 58°C +30x AT 60°C
Notch2 Exon 29	tttgcccagctgtaacctct	ggctccaggaaagacacaca	TD 65°C > 58°C +30x AT 60°C
Notch2 Exon 30	ctggagggctcaggttaatg	accagccagaaccatggag	TD 65°C > 58°C +30x AT 60°C
Notch2 Exon 31	gggttaatttgaatgtgtttgc	ccactgagccacctcatctt	TD 65°C > 58°C +30x AT 60°C
Notch2 Exon 32	cagggacacctcatttggag	tcctctcagaagggtagcaag	TD 65°C > 58°C +30x AT 60°C
Notch2 Exon 33	tgacctcatgctgctctctg	agagagacactgctgtgagcata	TD 65°C > 58°C +30x AT 60°C
Notch2 Exon 34.1	gaggaagcacttaggaaaagca	atgtggtggtgggatagcag	TD 65°C > 58°C +30x AT 60°C

(*Continued*)

	Forward primer	Reverse primer	PCR protocol
Notch2 Exon 34.2	gactctatcccccgtcgatt	tggcacaatggtctgagcta	TD 65°C > 58°C +30x AT 60°C
Notch2 Exon 34.3	cttatcccaaaaggcagcat	ctgcgtcctcccctaaaact	TD 65°C > 58°C +30x AT 60°C
Notch2 Exon 20–21	gaaggcgtccctttctttct	cggatgaactcagtagccagt	TD 65°C > 58°C +30x AT 60°C
Notch2 Exon 27–28	cagacctgacccttcgtttc	atgcccatctgctcatctct	TD 65°C > 58°C +30x AT 60°C

AT = Annealing temperature; TD = Touch down, lowering Annealing temperature over 7 cycles

Supplementary Table S2: Antibodies used for immunohistochemical staining of quiescent stem cells

Target	Product number and company
Wif-1	EPR9385, Epitomics
Lrig1	AF3688, R&D
Dll-1	Ab10554, Abcam
